# Feature Counting Is Impaired When Shifting Attention Between the Eyes in Adults With Amblyopia

**DOI:** 10.3389/fnins.2021.674146

**Published:** 2021-05-20

**Authors:** Chuan Hou, Gabriela Acevedo Munares

**Affiliations:** Smith-Kettlewell Eye Research Institute, San Francisco, CA, United States

**Keywords:** amblyopia, strabismus, selective attention, feature counting, interocular suppression

## Abstract

**Background:**

Feature counting requires rapid shifts of attention in the visual field and reflects higher-level cortical functions. This process is drastically impaired in the amblyopic eye of strabismic amblyopes. In this study, we hypothesized that feature counting performance in anisometropic and strabismic amblyopes is further impaired when shifts in attention is required between the eyes.

**Materials and Methods:**

Through a mirror stereoscope, highly visible Gabor patches were presented to the same eye within a block or randomly presented to the left eye or to the right eye with an equal probability within a block. The task was to report the number of Gabors (3 to 9) as accurately as possible. Counting performance was compared between the amblyopes and the normal-vision observers and between the viewing conditions (shifting attention between the eyes versus maintaining attention in the same eye).

**Results:**

When attention was maintained in the same eye, the amblyopic eye of both anisometropic and strabismic groups undercounted the number of Gabors, but achieved near-perfect performance with their fellow eye, compared to normal-vision observers. In contrast, when shifting attention randomly to the left or to the right eye, the amblyopic eye further undercounted the number of Gabors. Undercounting was also found in the fellow eye of strabismic amblyopes, but was not in the fellow eye of anisometropic amblyopes. Performance in normal-vision observers did not differ between shifting attention between the eyes and maintaining attention in the same eye.

**Conclusion:**

Our data showed that the amblyopic eye of both anisometropic and strabismic amblyopes further undercounted features when shifting attention between the eyes, compared to when maintaining attention in the same eye. This suggests that the ability to quickly redirect attention, particularly under interocular suppression, is impaired in amblyopia. The fellow eye of strabismic amblyopes also undercounted features when shifting attention between the eyes. However, such fellow eye abnormality was not found in anisometropic amblyopes, suggesting that different patterns of visual deficits are associated with amblyopia of different etiologies. The inability to count multiple features accurately reflects dysfunctions of high-level cortices in the amblyopic brain.

## Introduction

Amblyopia, the leading cause of monocular vision loss worldwide, is a neurodevelopmental disorder of vision, affecting about 3% of the population (Holmes and Clarke, 2006). Amblyopia is commonly caused by misaligned eyes (strabismus), chronic optical blur due to unequal refractive error in the two eyes (anisometropia), or a mixture of both during early childhood. In addition to visual acuity loss in one eye and reduced stereopsis, individuals with amblyopia also exhibit diverse deficits, including deficits in illusory contour perception (Popple and Levi, 2000; Hou et al., 2014), contour integration (Hess et al., 1997; Kovacs et al., 2000; Chandna et al., 2001; Kozma and Kiorpes, 2003), global motion sensitivity (Simmers et al., 2003; Ho and Giaschi, 2006, 2007, 2009; Hou et al., 2008), object enumeration (Sharma et al., 2000; Li R. W. et al., 2011), attentional blink (Popple and Levi, 2008), object tracking (Ho et al., 2006; Tripathy and Levi, 2008), and decision making (Farzin and Norcia, 2011).

The diverse perceptual deficits in amblyopia described above are commonly reported in studies using tasks requiring high-level cortical functions. Therefore, such deficits are commonly considered as evidence of high-level cortical dysfunction in the amblyopic brain. For instance, Sharma et al. (2000) reported that the amblyopic eye of strabismic amblyopes is unable to count features accurately as the eyes of normal-vision observers, but the non-amblyopic fellow eye achieves near-perfect counting performance as the normal-vision eye (Li J. et al., 2011). The authors argue that the inability to count features accurately is due to high-level cortical dysfunction, but not due to the well-established limitations of low-level processing in the amblyopic visual system (Levi and Klein, 1985, 1986; Smith et al., 1997; Kiorpes et al., 1998). This is because their experiments ruled out low-level processing factors such as feature visibility, crowding, positional jitter, and abnormal temporal integration. Studies have also reported that feature counting requires rapid shifts of attention in the visual field (Egeth et al., 2008; Anobile et al., 2012). When the number of features to be enumerated is small (*N* < 5) and briefly presented, rapid, error-free performance is achieved through a process known as subitizing, which is thought to be “pre-attentive.” In contrast, when the number of features to be enumerated is larger than 4, performance is slow and subject to error (Balakrishnan and Ashby, 1992; Pylyshyn, 1994; Trick and Pylyshyn, 1994), as well as dependent on the higher visual pathways, in particular the parietal cortex (Sathian et al., 1999; Nieder et al., 2006; Nieder and Dehaene, 2009), a region known to be involved in visual attention (Bressler et al., 2008). Therefore, Sharma et al. (2000) suggests that the finding of inability to count multiple features are likely due to attention deficits from the visual input of the amblyopic eye. Indeed, a number of studies have also reported attention deficits in amblyopia, including attentional blink (Popple and Levi, 2008), object tracking (Ho et al., 2006; Tripathy and Levi, 2008; Ho and Giaschi, 2009; Secen et al., 2011; Chow et al., 2018), conjunctive visual search (Tsirlin et al., 2018), line bisection task (Thiel and Sireteanu, 2009) and feature counting under dichoptic viewing (Wong-Kee-You et al., 2020).

Behaviorally measured attention deficits in amblyopia are consistent with our previous EEG-source imaging study (Hou et al., 2016), in which we found that attentional modulation in visual cortex, including V1 and extra-striate cortex (hV4 and hMT+), from the amblyopic eye was degraded in adults with strabismic amblyopia. This degraded attentional modulation in V1 was also correlated with the magnitude of interocular suppression and the depth of amblyopia, suggesting that interocular suppression may play a role of attention deficits in amblyopia. Supporting this, in a previous study (Wong-Kee-You et al., 2020), we found that feature counting under dichoptic viewing was impaired in the amblyopic eye of people with amblyopia with greater impairment in strabismic amblyopes than in anisometropic amblyopes. Chow et al. (2018) reported that attention was biased to the non-amblyopic fellow eye of amblyopia with dichoptic multiple-object tracking tasks. This bias was only found in strabismic amblyopes, but not in anisometropic amblyopes. These studies imply that in the natural visual environment, which is binocular and elicits interocular suppression, more severe deficits in feature counting in amblyopia may be revealed, compared to that under monocular viewing (Sharma et al., 2000). This speculation is based on previous studies that interocular suppression is stronger in strabismic amblyopes than in anisometropic amblyopes (Holopigian et al., 1988; Harrad and Hess, 1992; Agrawal et al., 2006; Narasimhan et al., 2012), although not all studies have found this (Li R. W. et al., 2011).

In the current study, we hypothesized that feature counting performance is further affected under binocular viewing when shifting attention between the eyes in strabismic amblyopia, as compared to the performance under monocular viewing reported in the Sharma et al. (2000) study. To test our hypothesis, we used a variant of the Sharma et al. (2000) paradigm that was modified for our binocular approach. We replicated the experiment in the Sharma et al. (2000) study and compared the counting performance between monocular viewing condition when maintaining attention in the same eye and binocular viewing condition when shifting attention between the eyes. We expected to reveal further deficits in feature counting under binocular viewing condition in strabismic amblyopia, given the experimental environment of interocular suppression from our stimulus setting. In addition to include participants with strabismic amblyopia, we also included participants with anisometropic amblyopia in the current study. Given the different findings between anisometropic and strabismic amblyopia from previous studies (Levi and Klein, 1982a,b; Thiel and Sireteanu, 2009; Hou et al., 2014; Chow et al., 2018; Wong-Kee-You et al., 2020), we expected to reveal different patterns of feature counting deficits between these subgroups as well.

## Materials and Methods

### Participants

A total of 21 adults between 21 and 65 years old (mean ± SD, 43 ± 14) of both sexes (8 males) were recruited for this study from the San Francisco Bay Area via research advertisement. Among them, 13 participants had unilateral amblyopia with visual acuity (VA) equal or worse than 20/25 (0.1 logMAR) in the amblyopic eye, and VA equal or better than 20/20 (0 logMAR) in the fellow eye, measured with Bailey-Lovie LogMAR chart. Normal vision participants (also referred to as “Normal”; *n* = 8) had 20/20 or better VA in each eye. There was no significant difference (*p* = 0.89) in age between normal (mean ± SD, 43 ± 16) and amblyopic participants (mean ± SD, 44 ± 13). All participants were refracted under noncycloplegic conditions by one of the authors (CH), a pediatric ophthalmologist, before the experiments. Participants with amblyopia were classified into two subgroups. Anisometropic amblyopia (referred to as “Aniso”; *n* = 6) was defined as unequal refractive error between the two eyes of at least 1 diopter in any meridian and with no constant ocular deviation or history of strabismus surgery. Strabismic amblyopia (referred to as “Strab”; *n* = 7) was defined as a constant ocular deviation or a history of prior strabismus surgery with or without anisometropia. All strabismic participants were non-alternating strabismus. There was no significant difference (*p* = 0.20) in logMAR VA in the amblyopic eye between the anisometropic (mean ± SD, 0.48 ± 0.16) and the strabismic (mean ± SD, 0.61 ± 0.18) groups. Stereoacuity was measured with the Random-dot stereo butterfly (Stereo Optical CO., INC). Normals had stereoacuity of at least 40 arcsec. The dominant and non-dominant eye in Normals was determined using the hole-in-card test. The demographic information of the amblyopic participants is provided in Table 1. Anisometropic participants had measurable stereoacuity while most strabismic participants had non-measurable stereoacuity, as seen in Table 1. Participants who had congenital cataract, eccentric fixation (measured by a direct ophthalmoscope) and nystagmus or latent nystagmus (nystagmus that appears when covering one eye) were excluded from the study. The research protocol conformed to the tenets of the Declaration of Helsinki and was approved by the Institutional Review Board of The Smith-Kettlewell Eye Research Institute. Written informed consent was obtained before the start of the experiments.

**TABLE 1:** Clinical details of the participants with amblyopia.

				**Visual acuity (logMAR)**					
**Participant number**	**Diagnosis**	**Age**	**Gender**	**Fellow eye**	**Amblyopic eye**	**Stereoacuity**	**Deviation**	**History**	**Experiment**
1	A	22	M	−0.097	0.341	200′	Ortho	Patching Done	1
2	A	52	F	0.04	0.518	200″	Ortho	Patching Done	1 and 2
3	A	51	F	0	0.739	200″	Ortho	Patching done	1 and 2
4	A	49	F	0	0.301	70″	Ortho	Patching done	1 and 2
5	A	50	F	−0.2	0.498	800′	Ortho	Patching done	1 and 2
6	A	21	M	0	0.498	140″	Ortho	No patching	1 and 2
7	S and A	59	M	−0.04	0.836	n/a	XT 14, L/R 14, DVD	Surgery and patching	1 and 2
8	S	38	M	−0.097	0.341	n/a	XT 12, R/L 4	Patching done	1
9	S and A	34	F	0	0.518	n/a	XT 8	Surgery and patching	1 and 2
10	S and A	62	M	0	0.756	n/a	XT 4, R/L 20, DVD	Patching done	1 and 2
11	S and A	65	F	−0.02	0.518	2000″	XT 8	Surgery and patching	1 and 2
12	S and A	36	F	−0.097	0.538	n/a	ET 4	Surgery and patching	1
13	S and A	46	F	0	0.756	n/a	ET 14	Patching done	1 and 2

*A, anisometropic amblyopia; S, strabismic amblyopia; S and A, mixed strabismus and anisometropia. Deviation at near with correction is shown in prism diopters. DVD, disassociated vertical deviation; XT, exotropia. ET, esotropia; L/R, left-eye hypertropia; R/L, right-eye hypertropia. “n/a” indicates that stereoacuity was not measurable with Random-dot stereo butterfly (Stereo Optical CO., INC).*

### Stimuli and Experimental Design

We modified the Sharma et al. (2000) paradigm that was originally used for a monocular test for our binocular approach. The reason we used a variant of Sharma et al. (2000) paradigm was because this paradigm used highly visible Gabor patches and have ruled out low-level cortical feature deficits in amblyopia, such as feature visibility, crowding, positional jitter, abnormal temporal integration, and spatial scale shifts (Levi et al., 1994). We modified the Sharma et al. (2000) paradigm, in which we used the same display of Gabor patches that could be used for both monocular test (Experiment 1) and binocular test (Experiment 2).

### Experiment 1

In this experiment, we repeated the experiment 1 in the Sharma et al. (2000) study. The stimuli (Gabor patches) were tested under monocular viewing condition with attention maintained in the same eye within a block of trials, while the untested eye remained open and viewed blank gray screen. There were 6 normal-vision observers, 5 anisometropic and 5 strabismic amblyopes participated this experiment.

**FIGURE 1:**
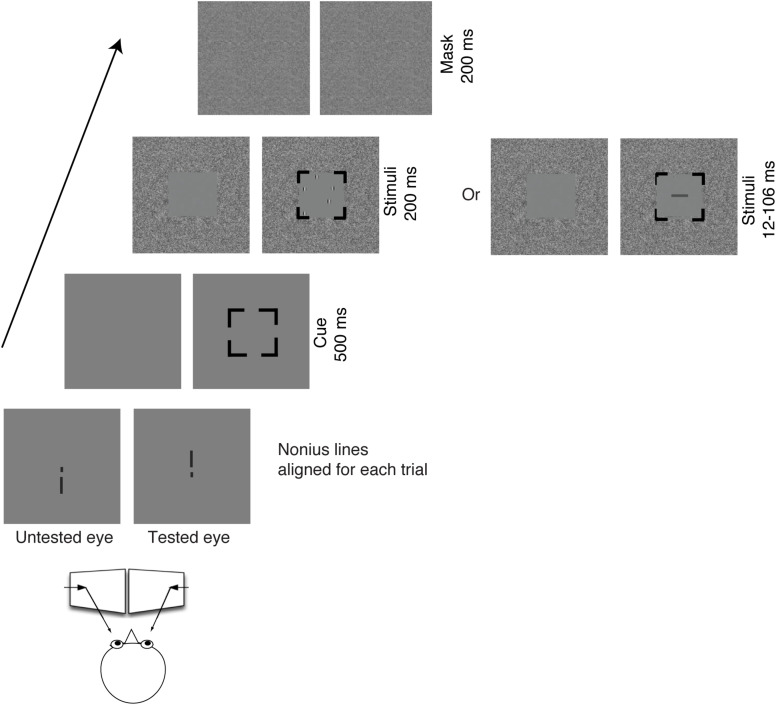
Illustration of the stimuli and the temporal sequence of a given trial viewing through a mirror stereoscope. In Experiment 1, the targets (Gabors) were presented to the same eye within a block. In Experiment 2, the targets (Gabors) were randomly presented to the left eye or to the right eye within a block. The task for Experiments 1, 2 was to count the number of Gabors. In Experiment 3, the target (a rectangle in horizontal or vertical orientation) was randomly presented to the left eye or to the right eye within a block with a wide range of display durations between 12 and 106 ms. The task for Experiment 3 was to report the orientation of the rectangle.

In the attended eye, a random array of Gabor patches was presented for 200 ms in the central visual field (5.6° square frame) surrounded by noise in the periphery (21° x 18° in the visual field), followed by a 200 ms noise mask. A 100% valid spatial cue (5.6° black square) displayed for 500 ms preceded the stimuli to the tested eye. This spatial cue was critical in Experiment 2 to guide attention to the tested eye when the stimuli were randomly presented to the left or to the right eye. Thus, the cue in Experiment 1 was to keep the same stimulus parameters as in Experiment 2. The stimuli and the temporal sequence of a given trial are illustrated in Figure 1. Participants were required to report the total number of Gabors (3-9) by pressing a button on the keyboard. In order to shorten the duration of the experiment, we skipped 4 Gabor patches in all experiments (including Experiments 1, 2). The spatial frequency of the presented Gabors was low (2 c/deg), as done in the Sharma et al. (2000) study, to compromise the poor visual acuity in the amblyopic eye of amblyopes. The contrast of Gabors was ≥ 25% for both eyes, but the contrast for the amblyopic eye was adjusted (matched) for equal visibility between the eyes (see below for details).

### Experiment 2

In this experiment, the stimuli (Gabor patches) were tested under a “binocular” viewing condition requiring attentional shifts between the eyes within a block. The targets were always viewed by the tested eye, while the blank screen was viewed by the untested eye. Here we used the term “binocular” as a comparison to the monocular test from a previous study (Sharma et al., 2000). Eight normal observers, 6 anisometropic and 7 strabismic amblyopes participated in this experiment. Among the participants in Experiments 2, 6 normal observers, 5 anisometropic and 5 strabismic amblyopes also participated in Experiment 1, which are marked in Table 1.

A random array of highly visible Gabor patches was randomly presented to the left eye or to the right eye with an equal probability within a block. A 100% valid spatial cue (5.6° black square) was displayed for 500 ms preceded the stimuli to guide participants’ attention to their tested eye. The task and stimulus parameters were the same as in Experiment 1.

### Experiment 3

In this experiment, the stimulus sequence and spatial cue were presented in the same manner as in Experiment 2, but the target was a single rectangle, instead of Gabor patches. Two normal observers, 2 anisometropic and 3 strabismic amblyopes, who also participated in Experiment 2, participated in this experiment.

Using a psychophysical procedure of constant stimuli, a single stimulus (a horizontal or a vertical rectangle ranging in size from 0.23° x 0.94° to 1.64° x 5.16°) was randomly presented to one of two eyes at 35% contrast for 12, 35, 60, 82 and 106 ms. The size of the rectangle was adjusted so that its orientation was correctly identified for more than 75% of the trials where the rectangle was presented for 60 ms monocularly to the amblyopic eye.

### Display and Procedure

Two Sony Trinitron Multiscan G400 CRT monitors, each with a frame rate of 85 Hz, were used to present the stimuli at a viewing distance of 85 cm. The stimuli were programmed in MATLAB (Mathworks, Natick, MA) with the Psychophysics Toolbox.

All participants were tested under their best-corrected vision. A practice block for each experiment was conducted to make sure the participant understood the task. Before the start of the trial, participants were required to adjust the mirror stereoscope to align nonius lines presented at the center of each screen. Participants repeated three blocks of trials and each block included 90 to 120 trials. The trials were self-initiated and the participants were required to respond as accurately as possible with no time limit and no feedback given.

### Contrast Match in the Two Eyes

All amblyopic participants adjusted the contrast in the two eyes for equal perceptual visibilities before the experiments. Through a mirror stereoscope, two horizontal sinusoidal gratings (3 c/deg, 2.5°) were separately presented with one in the upper visual field of the left eye and another in the lower visual field of the right eye. The participants were unaware of which eye saw which grating. The contrast in the fellow eye was fixed at 25%, while the contrast in the amblyopic eye was adjusted to match the perceptual visibility of the fellow eye. This procedure was repeated 3 times, and the average of all 3 contrast adjustments was defined as the balanced contrast for the amblyopic eye in Experiments 1, 2.

### Modeling

**FIGURE 2:**
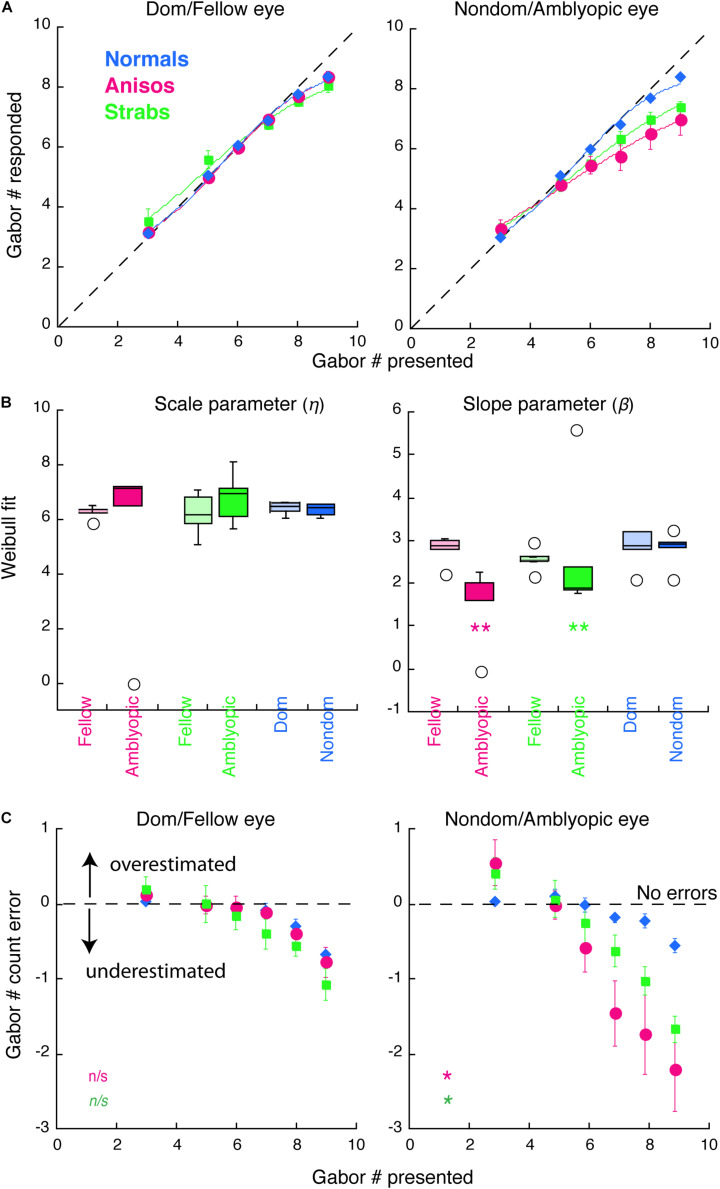
Counting performance in anisometropic and strabismic amblyopes and normal-vision observers when visual features were presented to the same eye within a block in Experiment 1. **(A)** Group mean of counting performance, in which the subjective estimates of the number are plotted as a function of the number of Gabor patches present. Colors denote the group. Error bars denote SEM. The dashed lines indicate 1:1 ratio between reported and displayed number of Gabors, representing correct estimates. Data below the dashed line indicate underestimates of the number of Gabors. Solid curves are the average model fits of Weibull function. **(B)** Comparison of the model fit results from individual participants with scale (η) and slope (β) parameters. **(C)** Group mean of counting errors from **(A)**. *, and ** denotes *p* < 0.025 (significance level by Bonferroni correction) and *p* < 0.01, respectively.

**FIGURE 3:**
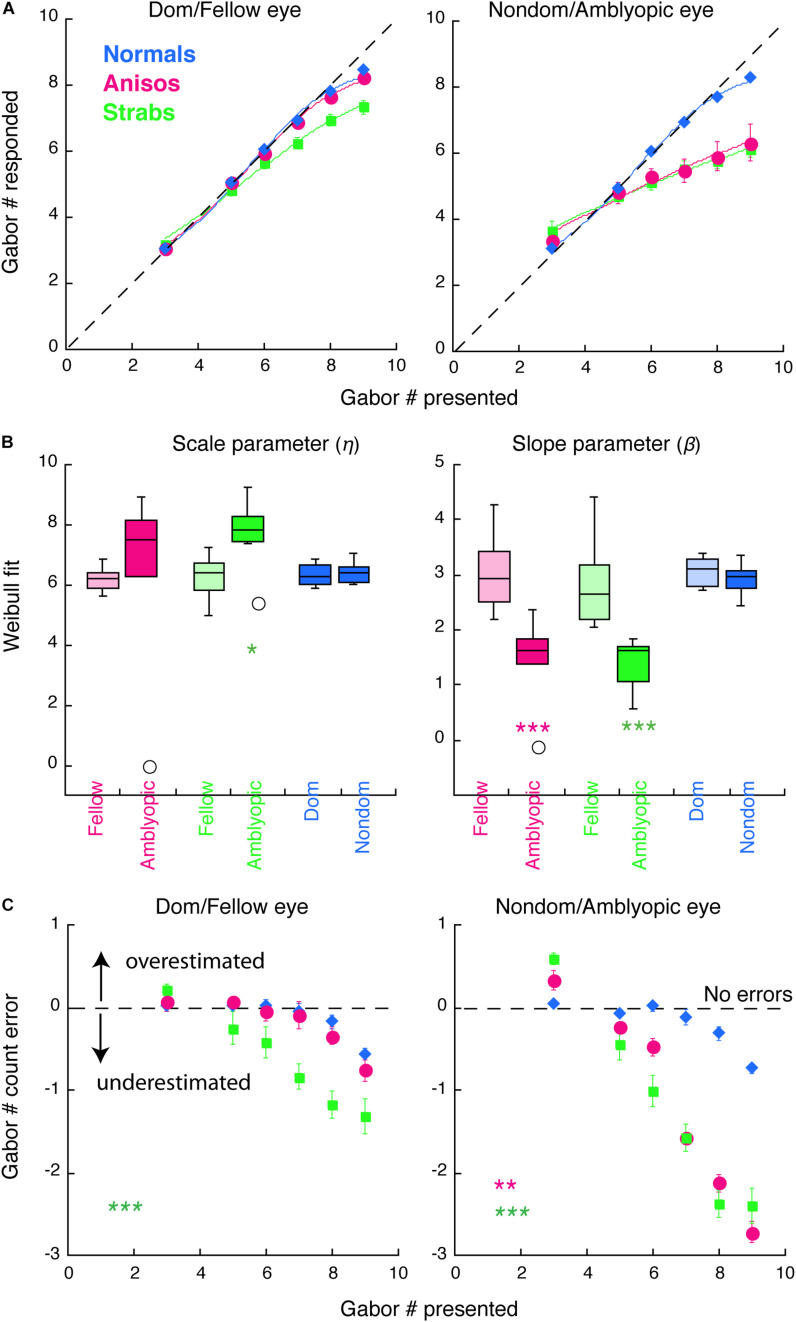
Counting performance in anisometropic and strabismic amblyopes and normal-vision observers when visual features were randomly presented to the left or to the right eye within a block in Experiment 2. **(A)** Group mean of counting performance. Colors denote the group. Error bars denote SEM. The dashed lines indicate 1:1 ratio between reported and displayed number of Gabors, representing correct estimates. Data below the dashed line indicate underestimates of the number of Gabors. Solid curves are the model fits of Weibull function. **(B)** Comparison of model fit results from individual participants with scale (η) and slope (β) parameters. **(C)** Group mean of counting errors from **(A)**. *, **, and *** denote *p* < 0.025 (significance level by Bonferroni correction), *p* < 0.01 and *p* < 0.001, respectively.

A Weibull cumulative distribution function with additional scaling coefficient and constant offset (equation 1) was used to fit the data as shown in Figures 2, 3. Fitting of these functions was done in KaleidaGraph to extract the scale (η) and shape (β) parameters.

(1) $$\text{y}=\left\{1-\exp\left[{\left(\frac{-\text{x}}{\eta }\right)}^{\beta }\right]\right\}\,\Delta_{N}+N_{0}$$

where η is a semi-saturation constant, β represents the slope of Weibull function, Δ_*N*_ is the amplitude scaling, and *N_0* is the constant offset. The coefficient of determination (R^2^) was used to assess goodness of fit of the model.

### Statistical Analysis

**FIGURE 4:**
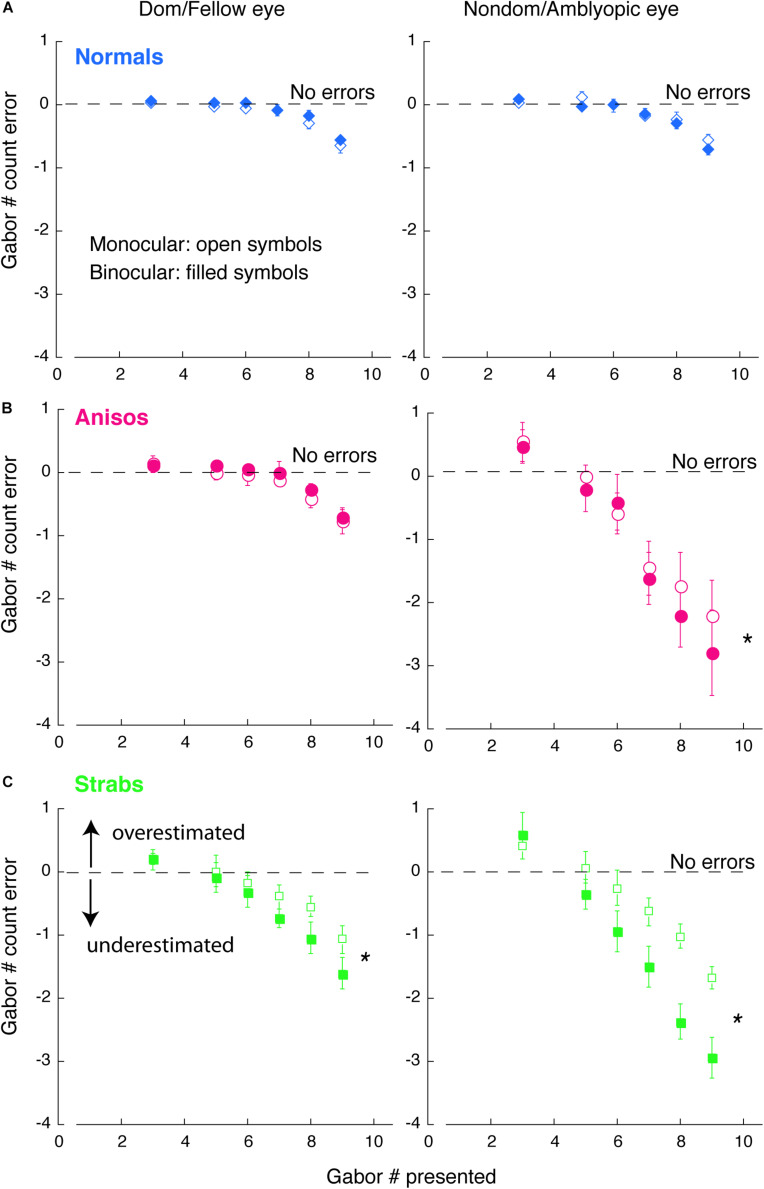
Comparison of counting errors between monocular and binocular viewing in each eye of **(A)** Normals, **(B)** Anisos, and **(C)** Strabs. Left column: Dom/Fellow eye; Right column: Nondom/Amblyopic eye. Open symbols: monocular viewing; Filled symbols: binocular viewing. The horizontal dashed lines indicate no counting errors. Data below the dashed lines indicate underestimates of the number of Gabors; and data above the dashed lines indicate overestimates of the number of Gabors. * denotes *p* < 0.025 (Bonferroni correction).

**FIGURE 5:**
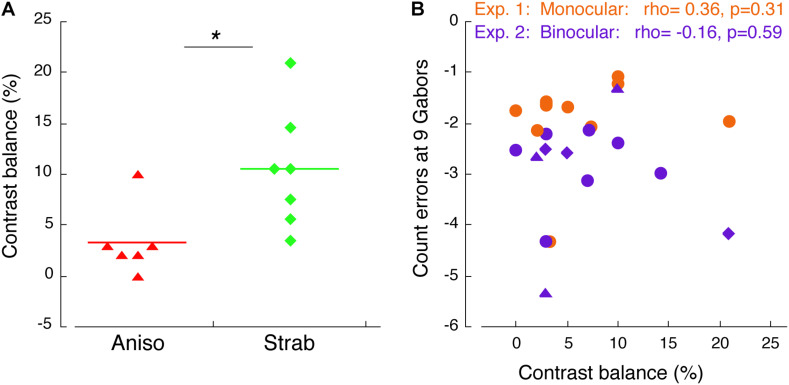
Correlation of counting performance and contrast balance. **(A)** Contrast balance between anisometropic and strabismic amblyopes. * denotes *p* < 0.05. **(B)** Correlation of counting error at 9 Gabors and contrast balance in Experiment 1 (orange) and Experiment 2 (purple). Triangles indicate anisometropic amblyopes and diamonds indicate strabismic amblyopes, who also participated in Experiment 3.

The primary analyses were conducted using a mixture of between- and within-subjects design ANOVA (Figures 2, 3) and a within-subjects design ANOVA (Figure 4). ANOVA was conducted in R. The Bonferroni correction was used to control the familywise error rate for repeated-measures ANOVA in each eye of the participants, in which the significance level was at 0.05/2 = 0.025. Significant differences in age between amblyopes and controls and visual acuity (logMAR) in the amblyopic eye between anisometropic and strabismic amblyopes were identified with the two-tailed heteroscedastic t tests. The significance of Weibull fit parameters between groups (Figures 2B, 3B) and significant differences in the contrast balance between anisometropic and strabismic amblyopes (Figure 5A) were identified with the one-tailed, Mann-Whitney Test for two independent sample. Correlation coefficient and its significance were calculated with Spearman’s rho with two-tails (Figure 5). Mann-Whitney Test and Spearman’s rho were conducted using the Real Statistics Resource Pack software (Copyright: 2013 – 2020, Charles Zaiontz)^1^.

## Results

### Experiment 1

#### Visual Features Were Presented to the Same Eye Within a Block

In this experiment, our goal was to replicate the experiment 1 in the Sharma et al. (2000) study where Gabor patches were presented only to one eye within a block. For simplicity, we refer to the amblyopic eye as “AE,” the fellow eye as “FE,” the dominant eye of Normals as “dom” and the non-dominant eye of Normals as “nondom” in the figures. Our data showed that the amblyopic eye of both the anisometropic and the strabismic group underestimated the number of Gabors, which reproduced the findings in the Sharma et al. (2000) study for strabismic amblyopes.

Figure 2 plots the counting performances for 3 groups (normal, anisometropic and strabismic groups). As seen in Figures 2A,C (left panels), all 3 groups, when using their dominant/fellow eye, were accurately able to estimate the number of Gabors until a set-size of 7, with errors emerging at set-sizes of 8 and 9 Gabors. In contrast, when using their amblyopic eye, the anisometropic and strabismic groups were only able to accurately estimate the number of Gabors until a set-size of 5, whereas accurate estimation was maintained in the normal group at a set-size of 7 when using their non-dominant eye. Since the enumeration of the small and the large set-sizes of Gabors depends on different neural mechanisms, we separated the participants’ performances into two Gabor groups. The Small Gabor Size group included trials with Gabor set-sizes of 3, 5 and 6, and the Large Gabor Size group included set-sizes of 7, 8 and 9. We expected to reveal counting deficits in amblyopia with the Large Gabor Size group, because their enumeration engages more attentional efforts than the Small Gabor Size group. An initial 4-factorial ANOVA (Group, Eye, Gabor Size Group and Gabor Set-size) revealed significant interactions among Group, Eye and Gabor Size Group (F_(2,12)_ = 4.83, *p* = 0.029). We therefore conducted sequential 1-factorial ANOVAs between groups for Large Gabor Size Group and Small Gabor Size Group to compare the performance of the amblyopic groups and the normal-vision group in each eye under higher levels of attention and lower levels of attention, respectively. For the Large Gabor Size trials, the results of the ANOVA revealed a significant difference only in the amblyopic eye of anisometropic amblyopes (F_(1,9)_ = 10.23, *p* = 0.011) and strabismic amblyopes (F_(1,9)_ = 10.23, *p* = 0.014), which underestimated the number of Gabors and made more errors (Figure 2C) as compared to the non-dominant eye of normal group. For the Small Gabor Size group trials, the results of the ANOVA revealed no significant difference between the eyes of amblyopes and the eyes of normal-vision observers (*p* > 0.05).

Furthermore, we quantified the counting performance by fitting the data with a variant of the Weibull cumulative distribution-function (see details in Methods). The model fits of the group mean for each eye and each group are shown with the solid curves in Figure 2A. The distribution of the data fits of individual participants are shown in Figure 2B. The goodness of fit (R^2^) across all participants and both eyes was 0.9956 ± 0.0077 (mean ± SD). In the left panel of Figure 2B, the scale parameter eta (η), which represents a semi-saturation constant, did not reveal significant difference between the eyes of both the amblyopic subgroups and the Normal group. However, the slope parameter beta (β) revealed a shallower slope in the AE of the anisometropic group (*p* = 0.0019) and the strabismic group (*p* = 0.0012) compared to the non-dominant eye of the normal group, indicating that performance in the amblyopic groups was impaired when using the amblyopic eye, specifically. Both amblyopic groups underestimated the number of visual features, as shown in Figure 2C. Notably, the fellow eye of the strabismic group achieved nearly perfect performance, as compared to the dominant eye of normal group (F_(1,9)_ = 5.08, *p* = 0.051). The results of the strabismic group are consistent with the findings in the Sharma et al. (2000) study. The amblyopic eye performance of the anisometropic group were similar to that of the strabismic group (FE: F_(1,8)_ = 2.57, *p* = 0.148; AE: F_(1,8)_ = 2.45, *p* = 0.156), which is the first report that the amblyopic eye of anisometropic amblyopes also undercounts visual features. These data analyses revealed that the amblyopic eye of both anisometropic and strabismic amblyopes underestimated features.

### Experiment 2

#### Visual Features Were Randomly Presented to the Left or to the Right Eye Within a Block

In this experiment, we expected to reveal further deficits in feature counting when shifting attention between the eyes in strabismic amblyopia, as compared to the deficits when visual features were only presented to one eye within a block in Experiment 1. We particularly anticipated that these further deficits would be observed in our strabismic amblyopia group, given that the stimulus design of the current experiment was binocular, which induces greater levels of interocular suppression.

#### Both Eyes of Strabismic Amblyopes Undercounted Features When Shifting Attention Between the Eyes

Figure 3 plots the counting performance of the 3 groups. As seen in Figures 3A,C (green symbols), the strabismic group, when using their fellow (left panels) and amblyopic (right panels) eye, performed worse in comparison to normal-vision observers and started to make errors at the set-size of 5 Gabors. In contrast, when visual features were only presented to one eye within a block in Experiment 1, only the amblyopic eye of the strabismic group performed worse, with impairments starting at the set-size of 6 Gabors (Figures 2A,C). An initial 4-factorial ANOVA (Group, Eye, Gabor Size Group and Gabor Set-Size) revealed significant interactions among Group, Eye and Gabor Size Group (F_(10,17)_ = 9.03, *p* = 0.002). Thus, we further conducted sequential 1-factorial ANOVAs between groups for the Large Gabor Size trials and the Small Gabor Size trials, to compare performance between the amblyopic groups and the normal-vision group, across each eye. For the Large Gabor Size trials, the results of the ANOVA revealed significant differences in the amblyopic eye of both anisometropic (F_(1,12)_ = 20.43, *p* = 0.001) and strabismic (F_(1,13)_ = 72.19, *p* < 0.001) amblyopes, in which the amblyopic eye underestimated the number of Gabors and made more errors (Figure 3C) compared to the non-dominant eye of normal observers. Surprisingly, the fellow eye of strabismic amblyopes also underestimated number of Gabors compared to the dominant eye of normal observers (F_(1,13)_ = 28.31, *p* < 0.001, Figure 3C left panel), while no significant difference between the fellow eye of anisometropic amblyopes and the dominant eye of normal-vision observers (F_(1,12)_ = 1.12, *p* = 0.311) was found. For the Small Gabor Size group trials, the results of the ANOVA revealed no significant difference between the eyes of amblyopes and the eyes of normal-vision observers (*p* > 0.05).

As done in the Experiment 1, we also quantified the participants’ performance by fitting their data with a Weibull function (Figures 3A,B). The goodness of fit (R^2^) across all participants and two eyes was 0.9787 ± 0.0834 (mean ± SD). The slope parameter beta (β) revealed shallower slope for the amblyopic eye of the anisometropic group (*p* < 0.001) and the strabismic group (*p* < 0.001), compared to the non-dominant eye of normal group. Impaired performance in the amblyopic eye of both amblyopic groups, were primarily due to underestimations of the visual features, as shown in Figure 3C. The scale parameter eta (η) revealed a significant difference between the amblyopic eye of the strabismic group and the non-dominant eye of normal group (*p* = 0.0103).

#### Comparison of Feature Counting Between Monocular and Binocular Viewing Condition

We wanted to look at the performance across monocular and binocular viewing. Since 10 amblyopes participated in both Experiments 1, 2, we were able to compare counting performance between monocular and binocular viewing conditions within subjects.

Figure 4 plots the comparison of counting errors in group mean between monocular and binocular viewing condition in each eye of the normal, anisometropic and strabismic groups. As seen in Figure 4B and C (red and green symbols), both the amblyopic eye of the anisometropic and the strabismic groups undercounted the number of Gabors under binocular viewing (filled symbols) starting at the set-size of 5 Gabors, while under monocular viewing (open symbols), both groups undercounted from the set-size of 6 Gabors. This difference in impairment across binocular and monocular viewing, suggests that feature counting is further affected when attentional shifts between the eyes is required, such as under conditions of interocular suppression. In addition, the fellow eye of the strabismic group also undercounted the number of Gabors, from a set-size of 6 Gabors under binocular viewing and a set-size of 7 Gabors under monocular viewing. These findings in the strabismic group suggest that with the natural viewing that is binocular, strabismic amblyopes encounters attention deficits, either with the amblyopic eye viewing or the fellow eye viewing. By contrast, the fellow eye of the anisometropic group estimated the number of Gabors similarly to the normal group. Moreover, the amblyopic eye of both the anisometropic and strabismic groups always overestimated when the number of Gabors to be enumerated was small (Gabor set-size = 3), as was reported in strabismic amblyopes in Sharma et al. (2000) study.

The observations described above were also confirmed with statistical analysis. An initial 5-factorial ANOVA (Group, Eye, Viewing condition, Gabor Size and Gabor Set-Size) revealed significant interactions among Eye, Viewing condition and Gabor Size (F_(1,13)_ = 4.78, *p* = 0.048). We further conducted 1-factorial ANOVAs for Viewing Condition with the Large Gabor Size group trials and Small Gabor Size group trials, to compare the performances between viewing conditions in each eye of the amblyopic groups. For the Large Gabor Size trials, the results of the ANOVA revealed a significant difference between the monocular and binocular viewing conditions in the amblyopic eye of both anisometropic amblyopes (F_(1,4)_ = 52.10, *p* = 0.002) and strabismic amblyopes (F_(1,4)_ = 17.72, p = 0.014), as seen in Figures 4B,C. These findings indicate that under binocular viewing condition, anisometropic and strabismic amblyopes exhibit greater impairments and underestimate visual features to a greater extent, compared to monocular viewing conditions. The ANOVA also revealed a significant difference between the monocular and binocular viewing conditions in the fellow eye of strabismic amblyopes (F_(1,4)_ = 21.12, *p* = 0.010), but no significant difference in the fellow eye of anisometropic amblyopes (F_(1,4)_ = 0.29, *p* = 0.620) was found. Both eyes of normal group had no significant difference between viewing conditions (*p* > 0.05). For the Small Gabor Size group trials, the results of the ANOVA revealed no significant difference between viewing conditions in each eye of the 3 groups (*p* > 0.05).

#### Correlation Between Feature Counting Performance and Interocular Suppression

We have showed that the amblyopic eye of both anisometropic and strabismic amblyopes further undercounted the number of Gabors when shifting attention between the eyes, as compared to when maintaining attention in the same eye, suggesting that redirecting attention between the eyes is impaired in amblyopia under experimental environment of interocular suppression. Previous studies used contrast difference between the two eyes (i.e., contrast balance) to represent interocular suppression (Li et al., 2013; Ooi et al., 2013). Since we matched contrast in the amblyopic eye to obtain an equal perceptual visibility to the fellow eye in both Exp 1 and 2, we used contrast balance to index the magnitude of interocular suppression for our amblyopic participants. Figure 5A depicts the comparison of the degree of interocular suppression between anisometropic and strabismic groups. Stronger interocular suppression was found in the strabismic amblyopes compared to the anisometropic amblyopes (p = 0.011). To determine whether there is relationship between counting performance and interocular suppression, in Figure 5B we plotted the participants’ feature counting errors from both Experiments 1, 2 at the set-size of 9 Gabors when using the amblyopic eye as a function of contrast balance. As seen in Figure 5B, there was no correlation between counting performance and interocular suppression in both Experiment 1 (*rho* = 0.36, *p* = 0.31) and 2 (*rho* = –0.16, *p* = 0.59).

### Experiment 3

#### A Single Task Was Randomly Presented to the Left or to the Right Eye Within a Block

Our Experiment 2 has demonstrated further counting deficits when shifting attention between the eyes in amblyopia, as compared to Experiment 1 when maintaining attention in the same eye (Figure 4). This was particularly evident for strabismic amblyopes, who exhibited deficits in both eyes. We also showed that interocular suppression (i.e., contrast balance) was stronger in the strabismic group than in the anisometropic group (Figure 5A). These results suggest that interocular suppression may play a role in these additional deficits. On the other hand, it is also possible that the greater deficits found for binocular viewing (Experiment 2), especially in strabismic amblyopes, could be due to deviation of the eyes, which would require longer stimulus display durations to deal with the eye misalignment. However, our previous feature counting study with a dichoptic viewing (Wong-Kee-You et al., 2020) found that increasing the display duration from 200 ms to 350 ms did not improve counting performance in both anisometropic and strabismic amblyopes. Therefore, Experiment 3 was conducted to address two questions: 1) whether deficits in amblyopia would still be observed under the condition, where a simple target alternates between the eyes but little to no attention is required; 2) whether strabismic amblyopes need longer display durations to perform this simple task compared to normal-vision observers. If strabismic amblyopes can perform a simple eye-alternating task that requires little to no attention as accurately as normal-vision observers at various display durations, then it is unlikely that the deficits in feature counting we observed in Experiments 1, 2 are a result of display durations being too fast. Instead, it would indicate that the deficits in feature counting are a result of impairments in attention and high-level cortical function.

**FIGURE 6:**
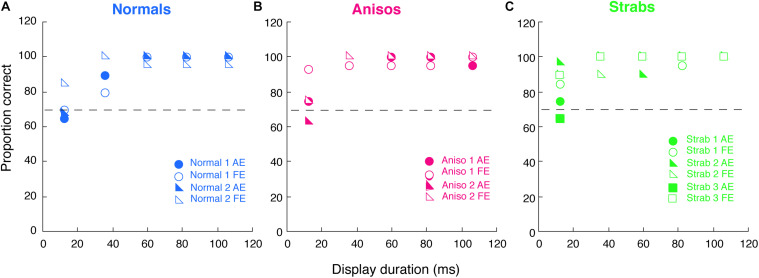
Proportion correct of a single target orientation discrimination as a function of the target display duration for 2 Normals **(A)**, 2 Anisos **(B)**, and 3 Strabs **(C)**, who also participated in Experiment 2. Open symbols, dom/fellow eye; filled symbols, nondom/amblyopic eye. Horizontal dashed lines indicate 75% correct.

Figure 6 plots the proportion correct of single target orientation discrimination as a function of the target display duration for 2 normal-viewing observer, 2 anisometropic and 3 strabismic participants who also participated in Experiment 2. These amblyopic participants exhibited counting deficits, which were marked in Figure 5B (i.e., anisometropic amblyopes were marked as triangles; strabismic amblyopes were marked as diamonds). The data from all 7 participants showed about 75% correct for the single target orientation discrimination at the shortest duration of 12 ms, and nearly 100% correct when the display was over 40 ms for both eyes. Notably, there were no differences between the two eyes in most display durations for all participants, as seen in Figure 6. Importantly, the strabismic group did not need longer display durations of the single target to perform this simple orientation discrimination task as accurately as the normal-vision observers. Thus, this experiment ruled out that the underestimates of multiple numbers of Gabors found in Experiments 1, 2 in strabismic amblyopes are due to the deviation of the eyes. Instead, the deficits are likely due to being unable to count multiple features accurately when the number of features is larger than 6.

## Discussion

Strabismic amblyopes have previously been found to undercount visual features when using their amblyopic eye under monocular viewing condition (Sharma et al., 2000). In the current study, we show that under binocular viewing condition where attentional shifts between the eyes are required, strabismic amblyopes further undercount when using their amblyopic eye. Notably, undercounting was found not only in the amblyopic eye, but also in the non-amblyopic fellow eye of strabismic amblyopes. Anisometropic amblyopes similarly undercounted visual features when using their amblyopic eye. However, when using their fellow eye, anisometropic amblyopes counted features as accurately as the normal-vision observers, whether attention was maintained on the same eye or shifted between the eyes.

### High-Level Cortical Dysfunctions and Attention Deficits in Amblyopia

During feature counting, small or large numbers of visual features are believed to recruit different neural mechanisms (Pylyshyn et al., 1994; Trick and Pylyshyn, 1994). Normal-vision observers can achieve error-free performance when counting up to 4 briefly presented visual features (Atkinson et al., 1976). This fast and error-free counting is thought to be ‘pre-attentive’. In contrast, the counting of briefly presented visual features at set-sizes above 5 requires spatial attention and the engagement of high-level cortical functions (Knudsen, 2007). In the current study, we used a variant of the Sharma et al. (2000) paradigm, which was originally used for monocular testing. The Sharma et al. (2000) paradigm had been tested for ruling out low-level cortical features, such as feature visibility, crowding, positional jitter, abnormal temporal integration, and spatial scale shifts in amblyopia (Levi et al., 1994). Therefore, in the current study the testing with a modified paradigm is believed to reflect high-level cortical functions, as claimed in Sharma et al. (2000) study. Our study found that both the amblyopic and fellow eyes of amblyopes, including both anisometropic and strabismic types of amblyopia, could achieve nearly accurate performance when counting from the Small Gabor Size group (Figures 2–4). This finding suggests that ‘pre-attentive’ processes, or the process with little attention are spared in the amblyopic brain. However, in both types of amblyopia, the amblyopic eye was unable to accurately count features when the set-sizes were in the Large Gabor Size group (i.e., large Gabor set-size of 7, 8 and 9), suggesting that attention processes are impaired in amblyopia, reflecting dysfunction of high-level cortices in the amblyopic brain.

### Relation of Selective Visual Attention and Interocular Suppression

When counting large set-sizes of Gabors (i.e., Gabor 7, 8, and 9) with shifting attention between the eyes (Experiment 2), additional counting deficits in amblyopia were found (Figure 4), as compared to those with maintaining attention in the same eye (Experiment 1). This is particularly evident for strabismic amblyopes, who exhibited deficits in both eyes. This finding suggests that the ability to quickly redirect attention between the eyes is further impaired in amblyopia, especially under the experimental environment of interocular suppression provided by our stimulus design with a binocular approach. People with strabismic amblyopia or anisometropic amblyopia usually suppress the visual percept from the deviating eye (strabismus) or from the higher refractive eye (anisometropia) to overcome diplopia (double vision) or visual blur. This long-term and chronic interocular suppression is believed to play an important role in amblyopic mechanisms (Jampolsky, 1955; Sireteanu, 1982; Holopigian et al., 1988). It has been reported that interocular suppression is stronger in strabismic amblyopia than in anisometropic amblyopia (Holopigian et al., 1988; Harrad and Hess, 1992; Agrawal et al., 2006; Narasimhan et al., 2012). We found this is also true in our amblyopic participants (Figure 5A). An fMRI study (Farivar et al., 2011) reported that the hemodynamic response function in response to amblyopic eye stimulation depended on whether the dominant fellow eye was open. When the fellow eye was open to a static pattern (not stimulated), the responses in the early visual cortex to amblyopic eye stimulation were reduced (suppressed) as compared to the responses when the fellow eye was patched and closed. This study demonstrated that interocular suppression exists once the fellow eye was open; no matter whether the fellow eye was stimulated or not. Consistent with this fMRI study, our findings revealed additional feature counting deficits in the amblyopic eye when redirecting attention randomly between the eyes, while the fellow eye was viewing a blank screen. Our results imply that there might be a relation between redirecting attention between the eyes and interocular suppression in the amblyopic brain. Such inability to redirect attention is unlikely related to poor visual acuity, because this defect was also found in the non-amblyopic fellow eye of strabismic amblyopes. Rather, it is likely related to selective attention deficits, particularly under interocular suppression. However, we did not find significant correlation between feature counting performance and interocular suppression (Figure 5B) in the current study. This might be due to our small sample size, or the measurement of interocular suppression that might not truly represent the magnitude of interocular suppression, because a certain number of strabismic amblyopes have a normal contrast sensitivity in their amblyopic eye (McKee et al., 2003). Thus, a future study with a better stimulus design is needed to reveal the correlation between feature counting performance and interocular suppression.

On the other hand, it also possible that shifting attention randomly between the eyes (Experiment 2) increased perceptual uncertainty between the eyes, as compared to the condition when attention was maintained in the same eye (Experiment 1). For example, individuals with amblyopia experience difficulty with spatial localiation tasks (e.g., Vernier tasks), and this is more evident in strabismic amblyopes than in anisometropic amblyopes (Levi and Klein, 1982a). When shifting attention between the eyes, this perceptual uncertainty could be more apparent for strabismic amblyopes under interocular suppression, as compared to that for anisometropic amblyopes. A previous study reported that perceptual uncertainty is a property of the cognitive system (Perea and Carreiras, 2012). It is not surprising to see more visual uncertainty in amblyopes than in normal-vision observers, as we have mentioned above regarding dysfunction of high-level cortices in the amblyopic brain. However, in our study, we were unable to know how much of perceptual uncertainty was created and how the interaction between interocular suppression and perceptual uncertainty was. Future studies are needed with well-designed experiments to manipulate and quantify the factors of interocular suppression and perceptual uncertainty in amblyopia.

### Different Pattern of Visual Deficits in Anisometropic and Strabismic Amblyopia

Our results showed that different feature counting deficits are associated with anisometropic versus strabismic amblyopia. When shifting attention between the eyes, the fellow eye of anisometropic amblyopes, like normal-vision observers, counted Gabors accurately up to 7 and started to make errors at 8. In contrast, the fellow eye of strabismic amblyopes was able to count features accurately at 3 Gabors, but started to make errors at 5 Gabors. Since we skipped 4 Gabors to reduce the exam duration, we were unable to show the performance at 4 Gabors. These findings are consistent with our previous dichoptic feature counting study (Wong-Kee-You et al., 2020). Wong-Kee-You et al. (2020) reported that when different numbers of Gabors were simultaneously presented to the left and the right eyes, participants with strabismic amblyopia exhibited greater deficits in feature counting in comparison to those with anisometropic amblyopia. The greater deficits in feature counting from strabismic amblyopes might have also confounded with their binocular disruption, since the tasks in that study engaged binocular fusion. However, the current study, which presented visual features in the same eye, still exhibited greater deficits in feature counting in strabismic amblyopes than in anisometropic amblyopes. The current study further confirmed a different pattern of visual deficits in anisometropic and strabismic amblyopia. The findings in the current study are also consistent with our previous electrophysiological studies that the fellow eye of strabismic amblyopes showed abnormal SSVEP responses to illusory contours (Hou et al., 2014), motion coherence (Hou et al., 2008) and selective attention (Hou et al., 2016), while the fellow eye of anisometropic amblyopes had normal SSVEP responses to illusory contours (Hou et al., 2014). The fellow eye deficits in strabismic amblyopes have also been reported in behavioral studies with position tasks (Vernier) (Levi and Klein, 1982a) and global motion-discrimination tasks (Giaschi et al., 1992; Simmers et al., 2003; Ho et al., 2005). These fellow eye deficits reported in previous studies commonly use tasks that primarily represent function of extra-striate or higher level cortices, and are more commonly found in strabismic amblyopia than anisometropic amblyopia (Hess and Demanins, 1998). The results, as we found in the current study, with different patterns of feature counting deficits in anisometropic and strabismic amblyopia strongly support the view that different patterns of visual deficits are associated with amblyopia of different etiologies.

In summary, in this study we demonstrated that the amblyopic eye of both anisometropic and strabismic amblyopes were unable to count multiple visual features greater than 6 accurately, supporting the view of attention deficits and dysfunction in high-level cortex of the amblyopic brain. More importantly, we found that the performance of feature counting was further affected when shifting attention between the eyes in amblyopes, as compared to when maintaining attention in the same eye. Our findings suggest that the ability to quickly redirect attention, particularly under interocular suppression, is impaired in amblyopia. We also found different patterns of feature counting deficits in anisometropic and strabismic amblyopia, supporting the view that different patterns of visual deficits are associated with amblyopia of different etiologies.

## Data Availability Statement

The original contributions presented in the study are included in the article/supplementary material, further inquiries can be directed to the corresponding author/s.

## Ethics Statement

The studies involving human participants were reviewed and approved by the research protocol conformed to the tenets of the Declaration of Helsinki and was approved by the Institutional Review Board of the Smith-Kettlewell Eye Research Institute. The patients/participants provided their written informed consent to participate in this study.

## Author Contributions

CH designed research, performed research, and wrote the first draft of the manuscript. GA analyzed data and edited the manuscript. Both authors contributed to the article and approved the submitted version.

## Conflict of Interest

The authors declare that the research was conducted in the absence of any commercial or financial relationships that could be construed as a potential conflict of interest.

## Footnotes

1 www.real-statistics.com
